# Use of the palm *Euterpe edulis* martius in landscape units managed by migrants of German origin in Southern Brazil

**DOI:** 10.1186/1746-4269-9-47

**Published:** 2013-07-04

**Authors:** Lucas de Souza Milanesi, Nivaldo Peroni, Maurício Sedrez dos Reis

**Affiliations:** 1 Biologist, Universidade Federal de Santa Catarina (UFSC), Florianopolis, SC, Brazil; 2 Researcher at Departamento de Ecologia, Universidade Federal de Santa Catarina (UFSC), Florianopolis, SC, Brazil; 3 Researcher at Departamento de Fitotecnia, Universidade Federal de Santa Catarina (UFSC), Florianopolis, SA, Brazil

**Keywords:** Landscape management, Ethnoecology, German migrants, Atlantic Rainforest, Homegardens, Secondary forest

## Abstract

**Background:**

People influence their environments through the manipulation of landscapes and species. Human influence on the landscape may lead to the development of differentiated landscape units that originate from past use and may be related to the presence of certain species. This study investigated the presence of the palm *Euterpe edulis* and its current and past importance in landscape units established by a community of German descendants located in southern Brazil. The objectives of this study were to characterize the use of the species, to identify the importance of *E.edulis* for the German immigrant community, to identify past and current uses of *E.edulis*, to describe the historical use of the landscape, and lastly, to identify landscape units in which *E.edulis* is found.

**Methods:**

The researched community is composed of people of German descent residing in southern Brazil. A variety of research tools were used to achieve the objectives of the research. Semi-structured interviews and free-listings were conducted in all family units. The interviews focused on groups of people in the community who had current or historical connection with the species. Group workshops and guided tours were conducted to identify different landscape units. The data were analyzed using descriptive statistics, use-value index, citation frequency, salience index, and informant perception analysis.

**Results:**

Over the historical period studied, the community demonstrated changes with respect to economic activities. These changes are reflected in the transformation of the landscape. The species *E.edulis* was and still is very important for people in the community; its importance is reflected in its high use value, citation frequency and salience. The species is found within various landscape units in the community as well as in homegardens and in secondary forests.

**Conclusions:**

The landscape heterogeneity of this community is influenced by changes in economic activities and by the relationship with the conservation unit. Landscape units resulting from this relationship may be identified. The species *E.edulis* is found within these landscape units and is integrated into the livelihood of the community.

## Background

The processes related to environmental modification, resource use, land conversion from forests to agriculture and regeneration of natural vegetation are complex. These processes are determined by ecological, social, economic, cultural and political variables that act on various spatial and temporal scales [1,2]. A migration event of human populations to a new environment involves processes of adaptation that have implications for native and non-native plant species and for landscape characteristics and structure at different intensities [3]. These aspects are particularly important in countries such as Brazil, the history of which is marked by inter-ethnic and cultural influences of migrant peoples that have mixed with indigenous people. Human influence on the landscape can occur at various levels, either through the manipulation of plant species populations or the manipulation of landscape components and abiotic factors, including ecosystem processes [4,5]. Thus, studies conducted on different spatial and temporal scales can be useful in understanding the human factors that influence the distribution and abundance of a plant species [5-7].

Studies that focus on landscape changes do not always clearly link the family unit level as a specific scale for analysis with actions that have influenced plant populations. However, focusing on this analysis level allows the understanding of processes that are directly related to livelihoods and natural resource demands, such as the use of plant genetic resources, e.g., domesticated plant species, species not used by humans, or species with the potential for use [8].

Research on the relationship of people to plant species at a landscape level associates changes in soil use and cover over time with economic phenomena, migration to urban centers, globalization, and demography [6,9-14]. These events, which are triggered by human action, can permit the recognition of historical use of some detailed landscape patches defined as landscape units or ecotopes [15,16]. Thus, landscape units can be defined as ecotopes and can be classified with respect to their history, specific use and management, biotic and abiotic characteristics, cultural meaning and the possession of an indigenous name or names that originates from the migrant peoples’ language [15,16]. Ethnoecological studies focused on plant species generally assess local ecological knowledge, species use, and social relations with the species and can be very useful in explaining the characteristics of landscape units or ecotopes [15-19].

The integration of landscape ethnoecology and species-level studies permits associations to be made between current and historical landscape forms that determine distinct landscape units and the use of plant genetic resources at specific locations [20]. Several species have been used in this approach. Palms (Arecaceae) are good examples because they possess both material and immaterial importance for local populations in the neotropics [21,22]. Moreover, South America is a center of richness and diversity of this plant family [23]. The uses of palms are diverse and include use as fibers, food, and tools for construction as well as medicinal and religious uses. The relationship between human populations and species within this family is widely documented, ranging from tolerance to intentional cultivation, and has resulted in the domestication of some species [21,24,25]. Documented uses for palm species vary from subsistence to trade in local, regional and even international markets [6,21,26].

The species *Euterpe edulis* is distributed from northeastern to southern Brazil and occurs in several ecosystems in the Atlantic Forest, ranging from rain forests to seasonal forests [27]. The relationships of people in the Brazilian territory with the palm *E.edulis* have been documented since the pre-colonial period [28]. During the sixteenth century, at the beginning of the Portuguese occupation of Brazil, references were made to the use of this species [29]. The use of *E.edulis* has persisted over time. However, its use for palm heart extraction increased progressively from the 1930s. This use involves cutting the entire plant to remove its apical meristem, which is used as food to supply consumer markets. In the 1970s, many populations of the species were already severely depleted due to commercial exploitation [27,30]. In 2008, the species was included on the official list of endangered species in Brazil [31]. Currently, there are initiatives in Brazil to use the fruit of the species, a use that does not entail the death of the adult plant.

Studies on the use of *E.edulis* in the rainforest reveal the importance of the species and show that there is considerable local ethnoecological knowledge about the species [18,32-35]. Nevertheless, little is known of how past human actions have determined its use and its occurrence within landscape units or ecotopes; specifically, little is known of how the species was influenced by a community of German migrants who arrived in Brazil in the nineteenth century and still maintain cultural German characteristics of language and religion. The Brazilian-German descendants of this immigrant community have retained German as a second language while using Portuguese as a first language and have named plants and landscapes both in Portuguese and in German. In southern Brazil, German migrants colonized several municipalities located in the Atlantic Forest; however, little is known of their relationship with this environment or of the origin of the Portuguese-German names used to recognize, use and manage plants and landscapes.

The focus of this study is to understand landscape transformations that have occurred at the local level in a specific area of Brazil under the influence of a people of German origin who determined the existence of anthropogenic landscape units and to associate them with the use of *E.edulis*. The specific objectives of the study are to characterize the livelihoods of the German descendants, identify the importance of *E.edulis* to the German immigrant community, identify past and current uses of the species, describe the historical use of the landscape and identify landscape units in which the species is found.

## Methods

### Study area

**Figure 1:**
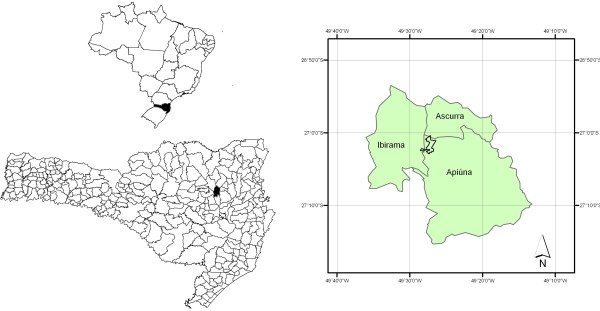
Location map the city of Ibirama in the state of Santa Catarina, Brazil.

The study area is located in Santa Catarina state in southern Brazil in the municipality of Ibirama in the Ribeirão Taquaras community, a community of German immigrants and descendants that is located in a rural area (Figure 1). The Xokleng indigenous group, who possessed nomadic habits and lived in the region for approximately 3000 years, initially occupied the Ibirama region [36]. At the end of the 19th century, colonization of the region by immigrants of German origin began; these immigrants established themselves near rivers and developed land for agricultural activities [37]. The German migrants came directly from Europe or from other colonies already established in Brazil, and they built houses and began cultivating crops primarily for subsistence [37].

The municipality of Ibirama has a population of 17330 and is divided into eight localities in addition to its urban center. The Ribeirão Taquaras community was selected for this study because the people of this community maintain cultural German traits and self-recognize as a German community and because of the presence of the species *E.edulis* in the landscape. The community is located approximately ten kilometers from the urban center of the municipality. Notably, the German dialect, which has been transferred down through approximately three generations, is still used within family units and at community gatherings by people who were born in the community.

Historically, the properties in Ribeirão Taquaras are small, and they still maintain this pattern. The current average property size is 25.7 hectares [38]. The landscape consists predominantly of a mosaic of secondary forests, agriculture and pastures [39,40]. The vegetation types present are montane rainforest at higher altitudes and submontane rainforest at lower elevations; both are part of the Atlantic Forest biome.

The community is located in the vicinity of a sustainable-use federal conservation unit (CU) (Ibirama National Forest) that was established in 1988 and consists of 519.23 ha. The CU has the objective of conserving biological diversity and promoting scientific research with a focus on native forest resources.

### Data collection

Initially, this study was submitted to the Ethics Committee on Human Research (CEPSH), of the Federal University of Santa Catarina (Certificate No. 944) and the Brazilian Institute of Environment and Renewable Natural Resources (IBAMA) through the System Authorization and Information on Biodiversity (SISBIO), which provided proof of registration (No. 24423-1) to collect botanical material, fungal and microbial which allowed us to start working in the field.

A census was conducted between September 2010 and January 2011 in which semi-structured interviews were conducted with each family unit. The interviews consisted of questions related to the economic profile and economic activities of the family, the uses of the property and the family’s relationship with the CU. The interviews were conducted with the householders; 65% (N = 65) of the family units in the community agreed to participate in the study.

To provide information on the importance of *E.edulis* in relation to other species utilized by the community, a survey on local species knowledge was conducted using the free-listings method [41]. The free-listings were carried out individually with people who agreed to participate after the census interview. A total of 91 free-listings were conducted.

During the free-listing, each participant was encouraged to cite plants in the use categories of food, medicine, and manufacturing; other uses spontaneously described by the informant were also recorded [41-43]. The ending of the free-listing was subject to saturation for that use category. Plants were cited by popular name in Portuguese or German and were collected for identification of variation in popular names referring to the same botanical species. Collection and identification were carried out according to the work of Poderoso *et al*. [44]. The specimens were deposited in the FLOR Herbarium at the Federal University of Santa Catarina.

Based on the census and free-listing results, informants who possess a current relationship with *E.edulis* and those who possess a historical relationship with *E.edulis* were identified. Specific interviews were then conducted with each informant group.

Among informants who currently use the species, the first category consisted of persons who cultivate the species in their homegardens, and the second category consisted of people who extract the fruit of the species to make juices and extract the palm for food. Twelve interviews were conducted with the informants in the first category regarding the uses of the plant, its origins, and the motivation for cultivation in the homegarden. To obtain information regarding the use of the plant for fruit collection, income generation from this activity, time of year when this activity is carried out, collection location, and the incentive for the activity, ten interviews were conducted with persons who only extract the fruit. These people were found during the free-listing by their ability to recognize the use of *E.edulis* fruits.

Individuals who had a historical relationship with the species provided information on the community’s transformations. The interview questions for this category were related to the changes seen in the landscape, its transformations, the environments recognized in the landscape (landscape units, ecotopes), economic activities, changes in the population living in the community, and changes in the use of *E.edulis* over time. To represent the category of older residents, informants who were at least forty years of age and had used their properties for at least twenty years were included because twenty years is the time period thought to be required for the establishment of a new population of *E.edulis* in the forest [45]. The interview sample on historical changes in the community consisted of eight people, and the duration of each interview was approximately one hour. These people were identified during the census and agreed to participate in the second stage of the research. The mean age of the informants was 63.3 years (standard desviation- sd = 12.3), their average time of residence in the community was 59.8 years (sd = 15.3) and minimal use of the property was 27 years, being that some informants had inherited property from their parents or grandparents. From the interviews with older residents, the landscape units were identified, and guided tours were performed to identify the locations of these landscape units in the forests and homegardens where *E.edulis* is found. Homegardens were defined as areas that have both native and exotic species, cultivated or not, and are located near the residence [46-48].

Information on the community’s historical changes obtained from individual interviews was triangulated with information generated by the use of two participatory research tools, a timeline and a historical graph [49,50]. The development of these tools was organized as a workshop with a group of residents over the age of 50. The participatory tools were used after all interviews were completed to confirm the historical information obtained from the older residents regarding the economic and social transformations and the changes in landscape that had occurred and the denomination and recognition of landscape units. The two participatory tools were used in a complementary way and allowed the identification of events in the history of the community since the 1950s. The timeline allows the recognition of key events within the last six decades, while the historical graph permits the quantitative evaluation of these events from the beginning of the period to the present by assigning an intensity scale with the use of easily recognizable symbols [49,50]. The “+” symbol was used to quantify an event; (+) indicated very little, (++) a moderate amount, (+++) a substantial amount and (++++) a large amount; the number zero (0) represented the absence or the end of an event. The transformation events analyzed using this tool were the presence of exotic tree plantations, the raising of livestock (cows and pigs), the cultivation of sweet manioc and tobacco crops, the transformation of native forest areas, the presence of secondary forests, the number of people who work in agriculture, sawmill activities with native plants, the presence of agroindustries that process sweet manioc, the number of palm-heart industries, and the quantity of *E.edulis* cultivated in homegardens. The workshop was mediated by one of the researchers, who recorded all of the information presented by the informants in a paper poster; another researcher took photographs and made notes on the workshop. At the end of the workshop, one poster that presented all of the information obtained was created. The session during which these methods were applied was about two hours in duration and included twenty-four people, both men and women.

### Data analysis

The census data were analyzed using descriptive statistics. The free-listing data were used to calculate the use value of each ethnospecies (VU) [51] and the salience index of *E.edulis*[42]. The ethnospecies are considered generic folk taxa [52].

All categories of use were considered in calculating the use value (VU) for an ethnospecies (VU = ΣU/N, where “U” is the number of citations for the ethnospecies and “N” is the number of informants). Use categories cited during the free-listing of ethnospecies that were cited by fewer than ten informants were included in the category “other uses”.

The salience index (Sj) was calculated only for the category of food plants because this type of use has been maintained for *E.edulis*. The salience index Sj considers the ranking of use citation for the ethnospecies and was calculated as Sj = Rj/nl, Sjtotal = ΣSj/n, where Rj is the ranking of the item on a list, nl is the number of items on the list, Sjtotal is the average number of the item’s position, and n is the total number of respondents. In addition to the salience index, the citation frequency of each ethnospecies was also calculated. To calculate the salience index, the software Anthropac 4.0 was utilized [53].

The analysis of the current and historical relationships of the community with *E.edulis* and of the historical transformations in the community and landscape was conducted using qualitative analysis of interviews and the participatory workshop described above in which the timeline and historical graph were used. The analysis was conducted by identifying response patterns related to each informant’s perceptions. The data obtained from these tools were organized into chronological tables, and consensual information was registered for each time interval to reconstruct the transformations that have occurred.

## Results

### Community characteristics

The family units interviewed totaled 118 men (54.6%) and 98 women (45.4%) with an average of 3.0 persons per family (sd = 1.3) averaging 35.4 years of age (sd = 20.5). The level of education is primarily the early grades of elementary school (47.4%).

The family unit income comes mainly from paid labor, which constitutes the primary source of income for 58.5% of the informants and is the occupation of most of the economically active persons in the community (51.2%). Many families combine employment activities in urban areas with subsistence farming, a trend that began in the mid-1980s with the weakening of the local agricultural economy [40]. The average age of economically active people whose work is dedicated exclusively to agriculture (mean = 42.9 years) is higher than that of people whose work is dedicated to non-rural occupations (mean = 32.9 years).

The areas of use are mostly private property (90.7%) with an average area of 17 ha (sd = 20.3); most are classified as small farms according to the Brazilian property classification [54]. Only 8 family units indicated that agricultural activities are their main source of income. Properties whose family incomes are associated with urban occupations were found to be smaller (mean = 13.3 ha) than properties belonging to family units whose incomes are primarily associated with agricultural activities (mean = 33.1 ha).

### Local historical changes

It was possible to reconstruct the history of the community from the 1950s based on information obtained from the interviews with long-time residents and from the participatory methods. The information obtained from individual long-time residents was confirmed during the workshop and is described below. All long-time residents interviewed perceived that changes related to the decrease in agricultural activities have occurred in the community since the 1950s. The extractive and agricultural activities that were historically performed in the community included dairy cattle and pig raising, planting of sweet manioc varieties (*Manihot esculenta*), timber extraction, and cultivation of native palm and tobacco plantations. Since the 1960s, planting of exotic trees such as pine (*Pinus* sp) and eucalyptus (*Eucalyptus* sp.) has acquired greater economic importance, with a significant increase between 1990 and 2000. Plantings of exotic trees have replaced some fields that once existed. The decrease in agricultural activities and the increased cultivation of exotic trees have been accompanied by a decrease in the number of people involved in rural activities. One important justification for the latter is the devaluation of agricultural products and the consequent difficulty of obtaining sustenance exclusively from agricultural activities. Thus, many planting areas have become plantations of exotic trees or regenerated secondary forest, which has expanded the forest cover of the community since the mid-1970s. In addition to the expansion of forest regeneration due to the abandonment of cultivated areas, another factor that has contributed to this process has been the regulation of the use of forest species due to restrictive legislation enacted in the 1990s. Furthermore, the creation of a federal conservation unit in the late 1980s expanded the mechanisms of prohibition, enforcement and punishment of farmers who use forest resources.

### Landscape units

**Figure 2:**
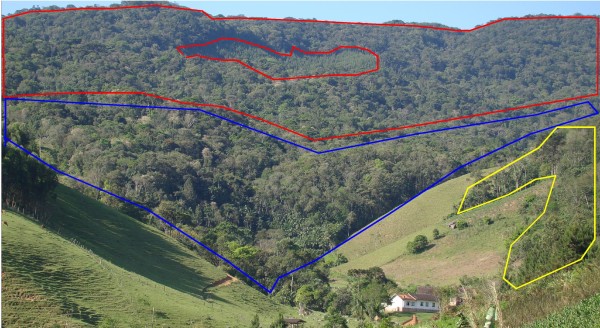
Units of landscape recognized for community of Ribeirão Taquaras. Red: “urwald”, blue: “aldacapavera”, yellow: “capavera”.

**Figure 3:**
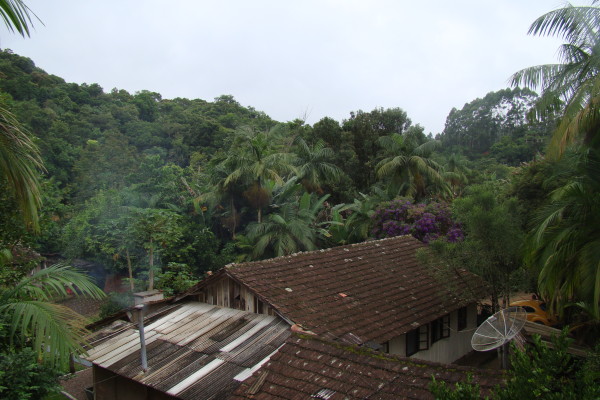
**The palm *E.edulis *grown in homegardens in the community.**

Information about landscape units was obtained through interviews with historical residents in workshops and through guided tours. The landscape units, forest environment and homegardens are those where *E.edulis* is found (Figures 2 and 3). Forest environments are distinguished and named in Portuguese and German according to different types of forests within the community. The community identifies the forest landscape unit (FLU) as “urwald” or “mato nativo” (native forest), which is defined as forest that has never been fully cleared for crops. This type of forest is recognized by the species that comprise it, for example *Ocotea* spp., by its physiognomy, such as the presence of large trees, and by the history of the area. Another recognized FLU is the “aldacapavera” or “altacapavera” in German and “capoeirão” in Portuguese, which is characterized by the presence of secondary forest of varying advanced ages. This FLU generally has a canopy height similar to that of a native forest, but its trees are smaller in diameter, and there is a predominance of initial or secondary pioneer species such as “jacatirão” in Portuguese or “jacatiron” in German (*Miconia cabussu* Hoehne, *M.cinnamomifolia* (DC.) Naudin), “cabroca” (*Myrsine coriacea* (Sw.) R.Br.), “licurana” (*Hyeronima alchorneoides* Allemão), “pau-jacaré” or “jacaré” (*Piptadenia gonoacantha* (Mart.) J.F. Macbr.), embaúba (*Cecropia pachystachya* Trécul.). Another FLU is “capavera” in German or “capoeira” in Portuguese, which is an intermediate between “capoeirinha” and “capoeirão”, comprising a lower canopy height with dominance of pioneer species such as “cabroca” (*M. coriacea*) and “jacaré” (*P.gonoacantha*). Another FLU is called “kleincapavera” in German and “capoeirinha” in Portuguese; this is an abandoned growing area two to three years old containing small trees and a reduced presence of *E.edulis* individuals. The word “capoeira” and its variations such as “capoeirinha” and “capoeirão” derive from the Tupi-Guarani expression [*caá* or *kaá* (forest)] + [*coêra* (which was)] and can be translated into English as “forest that existed”.

The FLU defines “aldacapavera”, “altacapavera”, and “capoeirão” as secondary forests resulting from a period of fallow after swidden cultivation. These landscape units expanded in area after reduction of community agricultural activities in the late 1970s.

The homegardens in Ribeirão Taquaras contain small populations or individuals of *E.edulis* along with other cultivated species. The information obtained from older residents and in the workshop reveals that homegardens containing *E.edulis* expanded in the community during the 1980s, whereas previously the species primarily occurred in forest environments in the area. Thus, landscape units on the farmers’ properties in which *E.edulis* is present are the “urwald”, “aldacapavera”, and “capavera” as described in German and homegardens that were reported in Portuguese as “quintais”.

### Current and past use of *Euterpe edulis*

Information on the current use of *E.edulis* was obtained through free-listings. High use value index (0.68, sixth position), salience index (0.287, tenth position among food species), and citation frequency (62.6%) were found for *E.edulis* (Tables 1 and 2). A total of 486 ethnospecies with some use were cited; of these, 192 were food plants. The most cited ethnospecies, in descending order, were sweet manioc (*M.esculenta*), orange (*Citrus sinensis),* tangerine (*Citrus reticulata*), jabuticaba (*Plinia trunciflora*), palm heart (*E.edulis*), cabbage (*Brassica oleracea*), collard (*B.oleracea*), cucumber (*Cucumis sativus*), banana (*Musa paradisiaca*) and guava (*Psidium guajava*). The index values obtained for *E.edulis* appear along with cultivated species and denote its importance for the people of the community.

**Table 1:** The use value of the first twenty ethnospecies that had higher values

**Ethnospecies**	**Species**	**Use value**
Orange, “laranja”	*Citrus sinensis* (L.) Osbeck	1.07
Corn, “milho”	*Zea mays* L.	1.02
Sweet manioc, “aipim”	*Manihot esculenta* Crantz	0.97
Tangerine, “tangerine”	*Citrus reticulata* Blanco	0.88
Guava, “goiaba”	*Psidium guajava* L.	0.85
palm-heart, “palmito”	*Euterpe edulis*	0.68
Banana, “banana”	*Musa paradisiaca* L.	0.67
“Jabuticaba”	*Plinia trunciflora* (O.Berg) Kausel	0.67
Mint, “hortelã”	*Mentha spicata* L.	0.66
collard greens, “couve”	*Brassica oleracea* L.	0.65
Eucalyptus, “eucalipto”	*Eucalyptus* sp.	0.63
Pine, “pinus”	*Pinus* sp.	0.62
Potato, “batata”	*Solanum tuberosum* L.	0.60
Cabbage, “repolho”	*Brassica oleracea* L.	0.56
Cucumber, “pepino”	*Cucumis sativus* L.	0.54
sweet potato, “batata-doce”	*Ipomea batatas* (L.) Lam.	0.51
“canela”	*Ocotea* sp.	0.51
Peach, “pêssego”	*Prunus persica* L.(Stokes)	0.51
Plum, “ameixa”	*Eriobotrya japonica* (Thunb.) Lindl.	0.49
Grape, “uva”	*Vitis vinifera* L.	0.47

**Table 2:** The first twenty ethnospecies for foods that had higher salience index and its citation frequency (%)

**Ethnospecies**	**Species**	**Frequency**	**Salience**
Sweet manioc, “aipim”	*Manihot esculenta* Crantz	86.8	0.593
Orange, “laranja”	*Citrus sinensis* (L.) Osbeck	84.6	0.560
Tangerine, “tangerine”	*Citrus reticulata* Blanco	79.1	0.538
Cabbage, “repolho”	*Brassica oleracea* L.	54.9	0.377
“Jabuticaba”	*Plinia trunciflora* O.Berg.	64.8	0.365
collard greens, “couve”	*Brassica oleracea* L.	54.9	0.330
Potato, “batata”	*Solanum tuberosum* L.	46.2	0.317
Cucumber, “pepino”	*Cucumis sativus* L.	53.8	0.309
sweet potato, “batata-doce”	*Ipomea batatas* (L.) Lam.	46.2	0.302
palm-heart, “palmito”	*Eutepe edulis*	62.6	0.287
Lettuce, “alface”	*Lactuca sativa* L.	45.1	0.281
Banana, “banana”	*Musa paradisiaca* L.	53.8	0.275
Peach, “pêssego”	*Prunus persica* L. (Stokes)	49.5	0.273
Guava, “goiaba”	*Psidium guajava* L.	52.7	0.270
Carrot, “cenoura”	*Daucus carota* L.	37.4	0.258
Grape, “uva”	*Vitis vinifera* L.	44.0	0.247
Beet, “beterraba”	*Beta vulgaris* L.	37.4	0.241
Corn, “milho”	*Zea mays* L.	41.8	0.226
Tomato, “tomate”	*Solanum lycopersicum* L.	37.4	0.207
Bean, “feijão”	*Phaseolus vulgaris* L.	36.3	0.207

According to long-time residents, *E.edulis* was used for various purposes in the past, including as stem anchors and in roof construction (1950s to 1980), as a fiber for making chairs, as fodder for cattle and as food (some long-time residents reported remembrance of its use as food during their childhood). Moreover, its importance at sporadic events, such as consumption of palm heart on festive occasions (birthdays, community celebrations) and its use to generate income for the improvement of equipment or for unexpected health expenses, was emphasized. Nine of the ten older interviewed residents mentioned that people within their family units extracted *E.edulis* on private properties and maintained some reproductive individuals in the forests locally known in Portuguese as “bagueiras”. The maintenance of some reproductive individuals was a rule in the past among community members that favored the *E.edulis* population. The theft of *E.edulis* individuals from these areas is seen as offensive and is a source of great dissatisfaction. Currently, within the forest areas there is extraction of the species only for consumption by family units. Nine historic residents and those present in the workshop perceived the increase in the number of palm individuals during the past 20 to 30 years as due to prohibition and regulation by the CU and the sowing of *E.edulis* seeds by farmers in the forest areas.

According to the long-time residents interviewed, the species occurs preferentially in the “urwald” and “aldacapavera” FLU. Four long-time residents indicated that the growth of the species in the “aldacapavera” is faster due to the high availability of light. Interestingly, farmers recognize different types of *E.edulis*, “white palm-heart” and “purple palm-heart”, which are found within different environments.

At the beginning of the 2000s, the community members began to gather and use the *E.edulis* fruit, calling the fruits “açaí”. Use of the fruit for this purpose can be explained by the influence of the successful use and commercialization of the Amazonian species *Euterpe oleracea* and *E.precatoria* in Brazil, which are known as “açaí” in the North. Interestingly, the use of “açaí” to refer to *E.edulis* fruit is borrowed from an Amazonian folk name that is currently used to refer to *E.oleracea* and *E.precatoria*. The gathering of *E.edulis* fruits took place primarily in homegardens because *E.edulis* individuals are smaller in size and there is less danger of falling while collecting the fruit. Managers of the CU and the state agricultural research organization encouraged this initiative by conducting courses for people in the community. However, the gathering of fruits has not yet become a source of income for the people of the community due to various factors that include its overlap with other activities performed by residents and difficulties in commercialization that reduce the income-producing potential of this activity for some farmers. A group of people maintains this activity, however, and has prospects of eventually increasing it in the future.

In addition to the use of the species to obtain palm heart and for pulp extraction for the production of “açaí”, the species was found to be cultivated in homegardens. Individuals of *E.edulis* were intentionally planted on the properties of five family units (42%) or favored after dispersal by fauna in four family units (33%). Three family units (25%) indicated that the occurrence of the species is due to planting as much as to dispersion by fauna. The motivation of the participants to let the species grow in homegardens included the use of the plant to obtain palm heart (cited by eight family units, 67%), its ornamental appearance (also cited by eight family units, 67%), and the attraction of animals or for shade, which was cited by two family units (17%).

## Discussion

Since the 1950s, the Ribeirão Taquaras community has experienced changes in local agriculture and in its primary modes of production and livelihood. Initially, the production systems in the community consisted of the cultivation of manioc, both sweet and bitter varieties, in swidden cultivation systems, and of livestock for milk. Afterwards, the logging industry expanded with the extraction of native species; subsequently, tobacco cultivation expanded in the community. These two activities have declined, primarily due to economic issues and environmental legislation [40,55], and the current trend in the region is toward the replacement of agricultural activities. The people who maintain agricultural activities tend to be older than those who have migrated to salaried jobs and industrial activities.

The economic weakening of rural activities in favor of industrial and urban activities resulted in a substitution of secondary farmland by forest. This is due to the change in production activities that has occurred over time [40,55].

The studied community demonstrates permeability between rural and urban areas, which makes it possible for people to continue to reside in the community even while modifying their economic activity. There seems to be no dichotomy between the rural and urban environments, and there is intense urban–rural interaction because rural activities for subsistence and commercialization still exist in the family unit despite the fact that the primary source of income comes from the urban space. Intense interaction between these two environments was also observed by Padoch *et al*. [56] in the Brazilian and Peruvian Amazon and by WinklerPrins [57] in the state of Pará in the Amazon.

The changes in economic activity that have occurred within the urban space have determined the changes in landscape within the community, principally the expansion of secondary forests. The expansion of secondary forests in abandoned agricultural areas is called forest transition and has been shown to occur in previous periods in developed countries [58,59].

Currently, Atlantic Rainforest fragments are composed of differently aged secondary forests [60]. For the state of Santa Catarina, 95% of approximately 25% of the remaining Atlantic Rainforest cover is secondary forest [61,62]. The natural resources of secondary forests are important to local communities, and abandonment of fields for forest regeneration does not make them idle and unproductive spaces [63-66]. The secondary forests within the community are an important environment for *E.edulis*, which was initially used in a way that sacrificed entire individuals for extraction of palm heart but now has a great potential for development in the extraction and use of fruits (“açaí”).

The perception of long-time residents of the Ribeirão Taquaras community is that there is a limited ability to use the forest and its species due to the creation of the CU. In addition to other factors associated with the abandonment of plantations in favor of forest expansion, the context of the CU may have favored this trend. The secondary forest in the study area, known locally as “aldacapavera”, varies in age, and *E.edulis* is present within it. The species may have been favored by the expansion of forest regeneration because the community had interest in its spread and because animals easily disperse the species between fragments of forest.

In addition to its presence in secondary forests, the species is found in homegardens. Homegardens are considered reservoirs of biodiversity because they have potential for in situ conservation of plant genetic resources and can contribute to household food security [46-48,67-70]. Homegardens are also important because they foster the exchange of knowledge and genetic resources between people, as well as experimentation by farmers. Thus, in addition to offering benefits to the family unit, homegardens contribute to the community’s collectivity [57,67,71,72]. In the community, homegardens are important for the diffusion of knowledge regarding the use of fruits from *E.edulis*. The transfer of a species from its naturally occurring environment to a cultivation environment is recognized as one method of plant domestication [48,67,73,74]. Cultivation of *E.edulis* is also found in other communities on the Brazilian Atlantic Forest coast [18] and in secondary forests in an agricultural community located in the state of São Paulo, Brazil [32].

The expansion of species cultivation near houses has occurred in the Ribeirão Taquaras community since the 1980s. The homegardens are variable with respect to the density and arrangement of *E.edulis*. The species is intercropped with other cultivated species and appears either spontaneously or intentionally in the space. Even if not intentional, the inclusion of the species within homegardens denotes a preference for its maintenance. The tolerance of *E.edulis* in homegardens is favorable for conservation; it also potentially reduces the use of the species within the forest environment, which may in turn cause a decrease in conflicts with managers of the CU [69,72,75]. The species function in homegardens belonging to family units is variable. In some cases, it is used for domestic consumption, home decoration, and animal attraction. Commercialization of *E.edulis* present in homegardens was not observed within any family unit; that is, it appears to be a resource that is used for family consumption. This finding differs from the results of other studies, some of which report an association of homegardens with the market [69,75,76]. The presence of palms is very frequent in homegardens and has been observed in studies conducted in the Brazilian Atlantic Rainforest in both *quilombola* communities and in urban communities [18,77], in urban communities in the Brazilian *cerrado*[78,79], in rural communities of the *caatinga*[69,72], in urban communities of the *pantanal*[75], in urban, rural, indigenous and Afro-descendant communities in the Amazon [57,73,74,76,80], where it is not always associated with markets.

The use of *E. edulis* in the community has changed over time; the species is currently maintained merely as an occasional food source, for the extraction of its fruits and for obtaining palm heart. The species is an important resource for the community, as shown by the fact that it received a high use value (VU), salience (Sj) and citation frequency. Ethnobotanical studies show significant values for species with high citation frequencies, salience index and use value; these high values reflect the importance of the food species for local communities throughout its range of occurrence, such as for *caiçara* communities, *quilombola*, and *açoriana* on the Brazilian coast [18,32-35,51,81].

Families that have a historical bond with the community report that the species was extracted from the forest on their property in cases of emergency. This reaffirms the importance of genetic resources as support for the equilibrium of family units [82]. However, this behavior is not uniform; other studies have described an exploitive relationship with the species in its distribution [83].

The species *E.edulis* is considered an important resource. In light of the economic interest associated with the species, several studies have highlighted a trend toward its depletion [84-86]. In this study, there was interest in cultivation of the species close to residents’ homes. The pattern of palm cultivation that occurs when use is suppressed to meet cultural needs was shown in Costa Rica for *Geonoma edulis*[87].

The community’s relationship with *E.edulis* was close and continuous; the species was used throughout the temporal interval analyzed in the study (since the 1950s). The highest frequency of its use was associated with the extraction of palm heart. However, new uses such as fruit extraction and cultivation of the species close to homes are emerging. The creation of new environments for the species, primarily secondary forests and homegardens, is associated with changes in the community. The community has maintained its cultural habits of religion and use of the German language since migration and has introduced the use and cultivation of *E.edulis* into its daily life, an occurrence that is reinforced by the value assigned to the species.

The permanent use of the species in the region makes it evident that the German community in this study considers *E.edulis* a very relevant species. *E.edulis* followed the trajectory of community transformation over time, and its use changed during the same period. At present, the species is embedded in various landscape units that have resulted from these historical transformations. The species is found in landscape units with varying degrees of human management and has the supremacy of both a native species (forests in different successional stages) and an exotic species (homegardens). The species receives a similar status to cultivated species, which are mostly exotic [44].

The results of this study show that species such as *E.edulis* that have high use potential and multiple applications have the ability to be incorporated into community habits and to establish themselves in a new place in such a manner as to receive a status equivalent to that of already domesticated species.

## Conclusions

The approach of this paper, which considers family unit characteristics as well as changes in the landscape and the community over time, makes it possible to understand the factors that have led to the current existence of heterogeneous landscapes. It was possible to relate the presence of *E.edulis* populations to these landscapes.

The studied community, which maintains German cultural habits (language and religion) from before its ancestral migration to Brazil, introduced *E.edulis* into the community through various uses and through cultivation and commercialization of the species. *E.edulis* is included in some landscape units identified in the community, and the importance given to the species contributes to the maintenance of the species population over time. Two landscape units in particular, homegardens and “aldacapavera”, are areas of importance for the use and conservation of the species, and a greater abundance of *E.edulis* is evident in these areas.

The identification of landscape units with different historical uses (“urwald”, “aldacapavera”, “capavera”, “kleincapavera” and homegardens) by the community allows the delineation of ecological studies that consider the human management of landscapes.

## Competing interests

The authors declare that they have no competing interests.

## Authors’ contributions

LSM and NP conceived, designed and coordinated of study, performed the field survey, carried out the analyses and prepared and drafted the manuscript. MSR participated in interpretation of results and drafted the manuscript. All authors read and approved the final manuscript.

## Authors’ information

LM. Biologist, Msc at the Universidade Federal de Santa Catarina (UFSC), Florianópolis, SC, Brasil. NP. Professor at Departamento de Ecologia e Zoologia, Universidade Federal de Santa Catarina (UFSC), Florianópolis, SC, Brasil. MSR. Professor at Departamento de Fitotecnia, Universidade Federal de Santa Catarina (UFSC), Florianópolis, SC, Brasil.
